# Cancer screenee cohort study of the National Cancer Center in South Korea

**DOI:** 10.4178/epih/e2014013

**Published:** 2014-08-06

**Authors:** Jeongseon Kim

**Affiliations:** Molecular Epidemiology Branch, Division of Cancer Epidemiology and Prevention, Research Institute, National Cancer Center, Goyang,Korea

**Keywords:** National Cancer Center, Cancer, Cohort, Korea

## Abstract

The Cancer Screenee Cohort Study was first established in 2002 by the National Cancer Center in South Korea to investigate all possible risk factors related to cancers and to expand biological specimen banking for the development of effective methodologies for cancer detection, diagnosis, and prevention. As of July in 2014, total 41,105 participants were enrolled in this cohort. Data were collected via questionnaire, clinical examination, cancer screening, and biological specimen testing including blood, urine, and exfoliated cervical cells. The highest incidence was found to be thyroid cancer, according to a nested case-control study that was linked to the National Cancer Registry information as of December 31, 2011. Case-control, cross-sectional, and cohort studies have been published using these data since 2009. Diet and nutrition was the most published topic, followed by genetics, hepatitis B virus and liver cancer screening, methodologies, physical activity, obesity, metabolic syndrome, smoking and alcohol consumption, and blood type. Evidence from the Cancer Screenee Cohort Study is highly anticipated to reduce the burden of cancer in the Korean population and aid in the detection, diagnosis, and prevention of cancer.

## INTRODUCTION

A cohort study is one type of observational research design performed over a specific follow-up period in which individuals with an incident disease of interest are compared with those who do not develop the disease. In South Korea, the Korean Multi-Center Cancer Cohort study (KMCC) was established in four geographically-defined urban and rural regions in 1993 and included adults in the general population-based over 35 [1]. This study was designed to investigate the relationship between exposures to specific factors (e.g., lifestyle) and the risk of cancers in the Korean population. Another population-based prospective cohort study, the Korean Genome and Epidemiology Study (KoGES), was initiated by the Korea Centers for Disease Prevention and Control in 2001 [2]. The KoGES was designed to determine genetic and environmental risk factors of major chronic diseases such as metabolic syndrome, diabetes, obesity, hypertension, cardiovascular disease, cancer, and others. To collect this large-scale epidemiological and clinical data as well as biospecimens, a national infrastructure for biomedical research was established. In South Korea, three major cohort studies have been established through KoGES: 1) a community-based cohort (in Ansan and Ansung cities, n=10,038) and a rural community-based cohort (n=28,000), 2) a health-examinee cohort (n=174,000), 3) a special cohort consisting of twins and families (n=3,300), Korean emigrants (n=3,600), immigrants after marriage (n=7,500), and members of the Asian Collaborative Cohort (n=4,000). It is expected that the findings of these studies will significantly decrease the burden of chronic diseases and increase the quality of life for Koreans [2].

Other developed countries such as the US, Japan, and Europe initiated large cohort studies throughout the last few decades to investigate the associations between risk factors and cancers. For example, the Japan Public Health Center-based prospective study on cancer and cardiovascular diseases was based in 11 public health centers and included a study population of more than 140,000 participants who had been followed from 1990 (cohort I) and 1993 (cohort II) [3]. This study was designed to provide evidence-based data for the prevention and control of cancer and cardiovascular diseases. In the US, the Nurse’s Health Study started in 1976, particularly focused on cancers and other chronic conditions [4]. In Europe, the European Prospective Investigation into Cancer and Nutrition was developed as a multi-center prospective cohort study throughout 23 centers in 10 European countries and included more than 500,000 participants [5]. This European study was designed to investigate the association between nutrition and cancer using dietary questionnaires collecting data on usual diet intake. In China, the Shanghai Women’s Health Study was established between 1996 and 2000 and recruited approximately 75,000 adult Chinese women from urban communities to investigate the major incidence of cancers and the influence of anthropometric risk factors such as smoking, alcohol intake, and others [6]. Similar to the women’s study, the Shanghai Men’s Health Study recruited a total of 61,500 adult Chinese men between 2001 and 2006 to investigate the influence of dietary and lifestyle factors on cancers and other chronic diseases [7].

Adapted after careful review of these previously implemented cohort studies, the Cancer Screenee Cohort Study was established in 2002 by the Center for Cancer Prevention and Detection at the National Cancer Center (NCC) in South Korea. The purpose of this study is to investigate possible risk factors related to cancers and to expand the banking of biological specimens for the development of effective methodologies for cancer detection, diagnosis, and prevention in the Korean population. In last decade, several influential cohort studies have been established and developed with the help of Korean government. In particular, the NCC collects some of the most representative data on cancer epidemiology, and it is highly anticipated that these data will help reduce the burden of cancer in the Korean population. Herein, this paper is to introduce the establishment and achievements of the Cancer Screenee Cohort Study at both national and international levels since 2002.

## STUDY PARTICIPANTS

From August 2002 till July 2014, 41,105 participants aged between 30 and 70 years were enrolled in this cohort study. Collected demographic data included the participants’ age, gender, marital status, education level, household income, job, smoking habits, alcohol consumption, and family history of cancer (Table 1). Participants who agreed to enroll in this cohort study were interviewed by trained personnel completed a health examination. Data were collected via questionnaire (e.g., past medical history), clinical tests (e.g., chest X-ray), physical examinations (e.g., blood pressure), cancer screenings (e.g., Pap smear), blood tests (e.g., cholesterol), urine tests (e.g., routine urinalysis), and other biological specimens (e.g., buffy coat) at the Center for Cancer Prevention and Detection at the NCC (Table 2). Trained personnel attempted to collect any missing information after the baseline collection via telephone. Until now, 44,234 participants have provided informed consent, 41,105 completed lifestyle questionnaire. Plasma were collected in 38,375 participants (vial n=159,816), buffy coat in 38,356 (vial n=74,385), red blood cell count in 38,358 (vial n=76,337), serum in 37,760 (vial n=141,553), whole blood samples in 9,097 (vial n=27,073). In addition, DNA was extracted in 19,209 participants, spot urine in 18,228, and uterine cervical exfoliated cells in 6,447 women.

## ETHICAL CONSIDERATIONS

The institutional review board of NCC approved this study in August 2002. All participants provided informed consent, were informed of their right to withdraw from the study at any time without penalty, and were informed that the purpose of this study is to detect, diagnose, and prevent cancer in South Korea.

## MEASUREMENT OF INCIDENT CANCER CASES

By matching resident registration numbers with participants, the incidence of cancer cases in the cohort was confirmed by linking with the Korea National Cancer Incidence Database of the Korea Central Cancer Registry [9]. The incidence of all cancer cases (n=1,498, male=824, female=674) as of December 31, 2011, was calculated by employing a nested case-control study. Table 3 shows the results of these calculations. Thyroid cancer had the highest incidence (n=507) in males (n=184) and females (n=323), followed by stomach (n=189, 128 males, 61 females), colorectum (n=155, 103 males, 52 females), lung (n=123, 83 males, 40 females), breast (n=107, 1 male, 106 females), prostate (n=99, 99 males), liver (n=67, 58 males, 9 females), kidney (n=28, 25 males, 3 females), bladder (n=27, 25 males, 2 females), gallbladder (n=17, 12 males, 5 females), and pancreas cancer (n=15, 14 males, 1 female).

## KEY FINDINGS AND PUBLICATIONS

Since 2009, the results of this study have been published in scientific journals. Published topics include diet and nutrition (14 publications) [10-23], genetic analyses (5 publications) [24-28], hepatitis B virus and liver cancer screenings (3 publications) [29-31], methodologies (3 publications) [32-34], physical activity (2 publications) [35,36], obesity (2 publications) [37,38], metabolic syndrome (1 publication) [39], smoking and alcohol consumption (1 publication) [40], and blood type (1 publication) [41]. Among these published studies, most employed a case-control study designs (17 publications) followed by cross-sectional (13 publications) and cohort study designs (2 publications) (Table 4). Published data using a cross-sectional design primarily used data from self-questionnaires to investigate the association between risk factors such as diet, physical activity, smoking, and alcohol consumption and chronic diseases (e.g., obesity, osteoporosis, metabolic syndrome, and colorectal adenomas). In addition, liver cancer incidence was associated with socioeconomic and lifestyle factors. Moreover, a nested case-control study on thyroid cancer is currently in progress and research on stomach, lung, and prostate cancer is expected to be completed for the next three to four years. However, cancers with a low incidence rate require active long-term.

Data from this cohort study will help determine risk factors of specific cancers in the Korean population. The findings of this study are also expected to be used for the development of a scientific model to determine the influence of interactions between genetic and environmental factors, and provide evidence-based preventive medicine at an individual level. Moreover, the findings of this study will aid in the design and evaluation of policies on a national level and in the exchange of scientific knowledge and manpower to coordinated future national and international studies.

## Figures and Tables

**Table 1.** Distribution of selected demographic factors in this cohort

Variables	Male (n=20,680)	Female (n=20,425)	Total (n=41,105)
Age groups (yr)			
30-39	2,786 (13.5)	2,566 (12.6)	5,352 (13.0)
40-49	7,575 (36.6)	7,764 (38.0)	15,339 (37.3)
50-59	6,603 (31.9)	6,885 (33.7)	13,488 (32.8)
60-70	3,716 (18.0)	3,210 (15.7)	6,926 (16.9)
Marital status			
Never married	627 (3.0)	663 (3.2)	1,290 (3.1)
Currently married	18,039 (87.2)	16,045 (78.6)	34,084 (82.9)
Widowed	166 (0.8)	1,025 (5.0)	1,191 (2.9)
Divorced/separated	372 (1.8)	792 (3.9)	1,164 (2.8)
Unknown	1,476 (7.1)	1,900 (9.3)	3,376 (8.2)
Education level			
Middle school or less	2,079 (10.1)	3,470 (17.0)	5,549 (13.5)
High school	5,574 (27.0)	7,666 (37.6)	13,240 (32.3)
College or more	11,257 (54.5)	6,929 (33.9)	18,186 (44.2)
Unknown	1,770 (8.6)	2,360 (11.6)	4,130 (10.0)
Household income (1,000 Korean won/mo)			
<200	2,256 (10.9)	3,182 (15.6)	5,438 (13.2)
200-400	5,783 (28.0)	5,271 (25.8)	11,054 (26.9)
≥400	9,778 (47.3)	7,413 (36.3)	17,191 (41.8)
Unknown	2,863 (13.8)	4,559 (22.3)	7,422 (18.1)
Occupation			
Professional, administration	6,677 (32.3)	1,870 (9.2)	8,547 (20.8)
Office worker	3,474 (16.8)	1,416 (6.9)	4,890 (11.9)
Sales, services	3,792 (18.3)	2,601 (12.7)	6,393 (15.6)
Agriculture, manual labor	3,255 (15.7)	1,005 (4.9)	4,260 (10.4)
Housewife	15 (0.1)	10,858 (53.2)	10,873 (26.5)
Unemployed, retired	1,670 (8.1)	540 (2.6)	2,210 (5.4)
Unknown	1,797 (8.7)	2,135 (10.5)	3,932 (9.6)
Smoking status			
Never smoker	3,825 (18.5)	16,376 (80.2)	20,201 (49.1)
Ex-smoker	7,883 (38.1)	664 (3.3)	8,547 (20.8)
Current smoker	7,945 (38.4)	895 (4.4)	8,840 (21.5)
Unknown	1,027 (5.0)	2,490 (12.2)	3,517 (8.6)
Alcohol consumption			
Never drinker	2,432 (11.8)	9,808 (48.0)	12,240 (29.8)
Ex-drinker	1,308 (6.3)	825 (4.0)	2,133 (5.2)
Current drinker	15,711 (76.0)	7,736 (37.9)	23,447 (57.0)
Unknown	1,229 (5.9)	2,056 (10.1)	3,285 (8.0)
Family history of cancer			
No	6,567 (31.8)	6,829 (33.4)	13,396 (32.6)
Yes	13,177 (63.7)	12,175 (59.6)	25,352 (61.7)
Missing	936 (4.5)	1,421 (7.0)	2,357 (5.7)

Values are presented as number (%).

**Table 2.** Description of health examination tests performed on cohort participants

Questionnaires/tests	Measurements
Questionnaire	Demographics, past medical history, family history of disease, occupation, medication use, cigarette smoking including environmental tobacco smoking, alcohol intake, food frequency questionnaire (food items/groups) or 3-day dietary records, female reproductive history
Clinical tests	Electrocardiogram, pulmonary function test, chest X-ray, body composition analysis (impedance), bone density (DEXA)
Physical measurements	Blood pressure, pulse, height, weight, waist circumference, visual acuity test
Cancer screenings	Esophagogastroduodenoscopy, mammography, cytology of cervix uteri (Pap smear), stool occult blood/colono-sigmoido-scopy/liver USG + aFP
Blood tests	Fasting plasma glucose, total cholesterol, high density lipoprotein cholesterol, low density lipoprotein cholesterol (direct measure), triglyceride, total protein, albumin, gamma-glutamyl transferase, aspartate aminotransferase, alanine amino transferase, creatinine, calcium, uric acid, complete blood cell count with differential count
Urine tests	Routine urinalysis
Biological specimens	Plasma, buffy coat, red blood cell, serum, whole blood, extracted DNA, random spot urine, uterine cervical exfoliated cells (women)

**Table 3.** The number of incident cancer cases^1^

Subsites	Men	Women	Total
Thyroid	184	323	507
Stomach	128	61	189
Colorectum	103	52	155
Lung	83	40	123
Breast	1	106	107
Prostate	99	-	99
Liver	58	9	67
Kidney	25	3	28
Bladder	25	2	27
Gallbladder	12	5	17
Pancreas	14	1	15
All cancers	824	674	1,498

1 Data were linked to the Korean National Cancer Incidence Database from the Korea Central Cancer Registry as of December 31, 2011 [8].

**Table 4.** List of published papers using data from the Cancer Screenee Cohort Study

	Study type [reference]		
	Cross-sectional	Case-control	Cohort
Diet and nutrition	[10-16]	[17-23]	-
Genetic analyses	-	[24-28]	-
Hepatitis B virus and liver cancer screenings	[29-31]	-	-
Methodologies	[32]	[33,34]	-
Physical activity	[35,36]	-	-
Obesity	-	-	[37,38]
Metabolic syndrome	-	[39]	-
Smoking and alcohol consumption	-	[40]	-
Blood type	-	[41]	-
